# Chromosome 11q13.5 variant associated with childhood eczema: An effect supplementary to filaggrin mutations

**DOI:** 10.1016/j.jaci.2009.10.046

**Published:** 2010-01

**Authors:** Gráinne M. O'Regan, Linda E. Campbell, Heather J. Cordell, Alan D. Irvine, W.H. Irwin McLean, Sara J. Brown

**Affiliations:** a Department of Paediatric Dermatology, Our Lady's Children's Hospital, Crumlin, Dublin, Ireland; b Epithelial Genetics Group, Division of Molecular Medicine, Colleges of Life Sciences and Medicine, Dentistry and Nursing, University of Dundee, Dundee, United Kingdom; c Institute of Human Genetics, Newcastle University, Newcastle Upon Tyne, United Kingdom; d Institute of Molecular Medicine, Trinity College Dublin, Dublin, Ireland

**Keywords:** Allergy, atopic dermatitis, atopy, inflammatory skin disease, skin barrier, AIC, Akaike information criterion, BIC, Bayesian information criterion, *FLG*, Filaggrin gene, *HRNR*, Hornerin gene, OR, Odds ratio, SNP, Single nucleotide polymorphism

## Abstract

**Background:**

Atopic eczema is a common inflammatory skin disease with multifactorial etiology. The genetic basis is incompletely understood; however, loss of function mutations in the filaggrin gene *(FLG)* are the most significant and widely replicated genetic risk factor reported to date. The first genome-wide association study in atopic eczema recently identified 2 novel genetic variants in association with eczema susceptibility: a single nucleotide polymorphism on chromosome 11q13.5 (rs7927894) and a single nucleotide polymorphism (rs877776) within the gene encoding hornerin on chromosome 1q21.

**Objective:**

To test the association of these 2 novel variants with pediatric eczema and to investigate their interaction with *FLG* null mutations.

**Methods:**

Case-control study to investigate the association of rs7927894, rs877776 and the 4 most prevalent *FLG* null mutations with moderate-severe eczema in 511 Irish pediatric cases and 1000 Irish controls. Comprehensive testing for interaction between each of the loci was also performed.

**Results:**

The association between rs7927894 and atopic eczema was replicated in this population (*P* = .0025, χ^2^ test; odds ratio, 1.27; 95% CI, 1.09-1.49). The 4 most common *FLG* null variants were strongly associated with atopic eczema (*P* = 1.26 × 10^−50^; combined odds ratio, 5.81; 95% CI, 4.51-7.49). Interestingly, the rs7927894 association was independent of the well-established *FLG* risk alleles and may be multiplicative in its effect. There was no significant association between rs877776 and pediatric eczema in this study.

**Conclusion:**

Single nucleotide polymorphism rs7927894 appears to mark a genuine eczema susceptibility locus that will require further elucidation through fine mapping and functional analysis.

Atopic eczema^1^ is a common inflammatory skin disease resulting in considerable morbidity in severely affected children.^2^ Genetic and environmental factors each contribute significantly to the etiopathogenesis of this disease, but our understanding of the role of genetic variation in atopic eczema remains limited, despite considerable research effort.^3^

An important breakthrough in understanding occurred with the identification of null mutations in the gene encoding filaggrin *(FLG)* on chromosome 1q21 as a major risk factor for atopic eczema.^4^ This insight has also served to focus attention on the role of epidermal barrier dysfunction as a key mechanism (perhaps the primary event) in eczema pathogenesis.^5^ *FLG* remains the most highly significant and widely replicated genetic risk factor for atopic eczema, with an estimated odds ratio (OR) of 3.12.^6^ However, even in the most severe case series, a maximum of 50% of cases carry *FLG* null alleles.^7^

The first genome-wide association study in atopic eczema has recently identified a novel susceptibility variant: a single nucleotide polymorphism (SNP) on chromosome 11q13.5.^8^ The same study reported an additional susceptibility SNP located within the hornerin gene *(HRNR)*, which encodes the protein hornerin, on chromosome 1q21, as well as again confirming the association of 4 prevalent *FLG* null mutations with atopic eczema in a European population.^8^

We aimed to investigate the roles of these 2 novel risk variants and their interactions with the well described *FLG* null mutations in a collection of Irish children with moderate-to-severe eczema.^1^

## Methods

### Study participants

A total of 511 unrelated children of self-reported Irish ancestry with moderate-to-severe eczema were recruited from tertiary referral pediatric dermatology clinics based at 1 center (Our Lady's Children's Hospital, Dublin). The diagnosis of eczema was made by experienced dermatologists according to the United Kingdom diagnostic criteria^9^ with disease severity scored according to the Nottingham Eczema Severity Score.^10^

A total of 1000 unselected control samples were obtained from the previously described population-based Trinity Biobank Control samples.^11^

Demographic and clinical data relating to the 511 Irish eczema cases and the 1000 individuals from the Irish control population are presented in Tables I and II.

The study was carried out in accordance with the Declaration of Helsinki Principles and approved by the Research Ethics Committee of Our Lady's Children's Hospital Crumlin (cases) and Trinity College Dublin (Trinity Biobank Control samples). All subjects or the subjects' guardians gave written informed consent.

### Genotyping methods

The 11q13.5 SNP (rs7927894) and the *HRNR* SNP (rs877776) were typed by the available predesigned Taqman allelic discrimination assays (Applied Biosystems, Foster City, Calif) according to the manufacturer's recommended protocols.

The 4 filaggrin loss-of-function variants that are most common in the Irish population (R501X, 2282del4, R2447X, and S3247X) were largely genotyped by using Taqman allelic discrimination assays, as described previously,^12^ or with modified and simplified protocols (Campbell and McLean, July 2009).

### Statistical genetic analysis

Allele and genotype frequencies were compared by using the χ^2^ test, the Fisher exact test, and logistic regression analysis, performed with the statistical analysis package STATA (StataCorp LP, College Station, Tex). Logistic regression models the log odds of disease as a linear function of variables encoding allele or genotype effects at the relevant locus or loci. For the allele model, a genotype variable coded as 0, 1, or 2 (according to the number of mutant alleles carried at a locus) was included in the regression equation. For the genotype model, a variable coding for homozygosity with respect to the mutant alleles was also included in the regression equation.

Tests for interaction were performed by including the main effects of rs7927894 and the relevant *FLG* mutation together with their interaction variables in the logistic regression equation.^13^ Because very few homozygous *FLG* mutant individuals were observed, we considered only a single main effect of *FLG* heterozygosity together with either a genotype effect at rs7927894 (resulting in 2 interaction variables, ie, a 2-*df* interaction test) or an allele effect at rs7927894 (resulting in a single interaction variable, ie, a 1-*df* interaction test).

The fit of different models to the data was compared via a Wald test (which assesses the significance of dropping a particular term from a regression model) and via calculation of the Akaike information criterion (AIC) and Bayesian information criterion (BIC). These 2 criteria measure the goodness of fit of an estimated statistical model in light of the number of observations and the number of estimated parameters. When using the AIC (or BIC) to compare models that have been fitted to an identical set of observations, the preferred model is the one with the lowest AIC (or BIC) value.

### Power calculation

Using the program QUANTO^14^ and assuming a population prevalence of 15%, we estimated that our sample had 71% power to detect the previously reported 11q13.5 (rs7927894) effect (OR, 1.22; allele frequency, 0.363) at 2-sided *P* value .05 (1-sided *P* value, .025). Our sample had only 43% power to detect the previously reported *HRNR* (rs877776) effect (maximum OR, 1.2; allele frequency, 0.158). We had >70% power to detect an interaction effect of OR of 1.64 or above between the 11q13.5 SNP (rs7927894) and *FLG* assuming a combined *FLG* mutation frequency of 0.078 and a *FLG* OR of 3 (at the lower end of data from comparable studies published previously).^15^

## Results

### Replication of the 11q13.5 locus association but not the *HRNR* association

Genotyping results for the 11q13.5 SNP (rs7927894), the *HRNR* SNP (rs877776), and the 4 *FLG* null mutations, R501X, 2282del4, S3247X, and R2447X,^12,16^ are shown in Table III, in addition to their corresponding allele frequencies. The *FLG* null mutations each showed strong and significant association with pediatric eczema in this case/control study when analyzed individually and as a combined null genotype (Fisher exact test, *P* = 1.26 × 10^−50^). The rs7927894 SNP also showed a significant association with eczema (χ^2^ test, *P* = .0025), but the rs877776 SNP was not significant (χ^2^ test, *P* = .318).

The results of logistic regression analyses to investigate the OR of disease are shown in Table IV. Separate analyses performed using allele-based and genotype models demonstrated the overall OR when allele frequencies were compared between case and control groups (allele model) and the distinct ORs relating to the heterozygotes and homozygotes for each genetic variant and the combined *FLG* null genotype (genotype model).

Tests comparing the fit of the genotype versus the allele models are shown in this article's Table E1 in the Online Repository at www.jacionline.org. The OR associated with the rs7927894 SNP was estimated at 1.27 by using the allele model; the genotype model gave an OR of 1.27 for heterozygote individuals and 1.63 for homozygotes (Table IV). The genotype model did not fit significantly better than the allele model (*P* = .95; the allele model had lower AIC and BIC). In comparison, the combined *FLG* null genotype showed an OR of 5.81 (95% CI, 4.51-7.49) by using the allele model, 5.23 (95% CI, 3.96-6.91) for heterozygotes and 140 (95% CI, 19.18-1021) for homozygotes, in keeping with semidominant inheritance. However, the low number of homozygous individuals meant that the difference between the genotype and allele models was not statistically significant (*P* = .12), although the genotype model did give the lower AIC, suggesting that it might be preferred over the allele model.

### The 11q13.5 and *FLG* loci are independent and multiplicative

Additional analyses performed to test the statistical significance of the rs7927894 SNP having controlled for the presence/absence of the strongly significant *FLG* null genotype indicated that rs7927894 still shows a statistically significant effect (*P* = .0025) with an OR of 1.22 (95% CI, 1.02-1.26). A mathematical model combining the effects of rs7927894 and *FLG* null genotype in a linear model fits the data significantly better (*P* = .0258) than allowing for *FLG* alone. Furthermore, tests for interaction between each of the *FLG* and rs7927894 risk alleles (Table E1, results based on the cross-classification of genotypes shown in this article's Table E2 in the Online Repository at www.jacionline.org) showed no evidence of statistically significant epistatic effects on the basis of either Wald tests from logistic regression or comparison of AIC and BIC values. Hence these data demonstrate that the risk of disease in individuals having *FLG* null mutations and risk alleles at rs7927894 is well fitted by a multiplicative model, with the OR multiplied by 5.21 for possession of a single *FLG* null allele (or 138 for possession of 2 *FLG* null alleles, although note the wide associated CI [18.92-1009.42]) and 1.22 times for each rs7927894 mutation (therefore 5.21 × 1.22 × 1.22 = 7.75 for an individual with a single *FLG* null allele and homozygous for mutations in rs7927894).

## Discussion

Many candidate gene studies in atopic eczema have been performed on relatively small case collections and have generated data that have not been replicated in further studies.^17,18^ However, in 1 of 5 reported microsatellite-based genome screens, the 1q21 locus was identified, and we have shown that a large component of this signal was a result of *FLG* null mutations.^4^ No additional loci have been identified with any certainty by other screening studies, and therefore the recent genome-wide association study, performed on German, French, and East European cases,^8^ was a very welcome addition to the field. As well as confirming the 1q21 locus and the hegemony of *FLG* mutations, this study generated other loci worthy of further investigation, of which 2 have been investigated in the current case-control study.

One SNP, rs877776, is located within *HRNR*,^8^ a gene encoding hornerin, an S100 fused-type protein that may be involved in epidermal barrier formation.^19^ Both *HRNR* and *FLG* lie within the epidermal differentiation complex, a dense cluster of genes on chromosome 1q21, many of which are thought to be involved in epithelial barrier function.^20^ Our 511 pediatric eczema cases include the discovery cohort for *FLG* loss-of-function mutations^4,16^ and are very well characterized for the prevalent and rare *FLG* null variants. In the current study, we could not replicate the *HRNR* association. However, this may simply be because our study is underpowered to detect this effect given its relatively low allele frequency and effect size. An alternative explanation is that the *HRNR* effect may be limited to the German/French/East European populations, or at least that it is not important in the Irish population. It is also possible that this SNP is in linkage disequilibrium with an as yet unidentified *FLG* null mutation in the study populations used for the genome-wide association study. Given that *FLG* mutations are highly population-specific,^21^ this seems a likely explanation. However, high-density SNP mapping, detailed sequencing of *FLG*, and examination of haplotypes in each of these populations would be required to confirm or refute this hypothesis.

The second novel SNP, rs7927894, is located on chromosome 11q13.5. We have confirmed an association of rs7927894 with eczema in a large, well phenotyped case collection of children with moderate-to-severe eczema. This case series represents a different population of white European ancestry than the discovery case series,^8^ as discussed in the previous paragraph. However, the ORs we obtained were very similar to the discovery cohort: the homozygous carriers of the risk allele show an OR of 1.63 (95% CI, 1.18-2.24) in the Irish pediatric eczema cases compared with an OR of 1.47 (95% CI, 1.29-1.68) in the European adult cases,^8^ providing replication evidence for this genetic association. Furthermore, the frequency of the rs7927894 risk allele is similar in our Irish population control group to that reported by Esparza-Gordillo et al^8^; 14.5% of the Irish controls were homozygotes, compared with approximately 13% in the European control population. The combination of such a prevalent risk allele with, it appears, a substantial OR illustrates the potential importance of this novel susceptibility locus in eczema.

The gene or gene product affected by the rs7927894 risk allele defined by this anonymous SNP remains to be identified and functionally characterized; rs7927894 lies in an intergenic region between 2 annotated genes, chromosome 11 open reading frame 30 (C11orf30) and leucine rich repeat containing 32 (LRRC32) (UCSC Genome Browser, Human Genome March 2006 assembly, http://genome.ucsc.edu). C11orf30 encodes the EMSY protein, which has been shown to bind the BRCA2 breast cancer susceptibility protein^22^ and may therefore play a role in epithelial differentiation. The second nearby gene, LRRC32 (also known as glycoprotein A repetitions predominant (GARP), has recently been shown to be a cell surface molecule expressed on regulatory T cells,^23^ and may therefore be a more likely candidate for atopic eczema susceptibility, given the important role of T-cell–mediated inflammation in atopic eczema.^24^ Although rs7927894 is located in the same haplotype block as C11orf30,^8^ providing tentative genetic evidence that this is the causative gene, it is possible that nearby genes such as LRRC32 may be regulated by sequences within this haplotype block. However, it is also possible that this SNP, or more likely, causative variants in linkage disequilibrium with it, may be involved in long-range control of more distant genes, well removed from either C11orf30 or LRRC32. Defects in noncoding long-range control elements, in many cases acting over distances spanning many intervening genes, are an emerging disease mechanism.^25^ Finally, it should be noted that there are some expressed sequence tag clusters close to rs7927894 (UCSC Genome Browser) that may represent further uncharacterized candidate genes.

Intriguingly, rs7927894 has also been identified as a susceptibility factor for Crohn disease.^8^ Thus, beyond skin-specific physical barrier genes, genes having an effect on susceptibility to epithelial inflammation may have similar functions in diverse inflammatory diseases affecting both skin and the gastrointestinal mucosal epithelia. There is limited evidence of an increased prevalence of atopic eczema in children with inflammatory bowel disease, and this cannot be explained by a differential prevalence of *FLG* null mutations.^26^ Although currently highly speculative, it is tempting to suggest that the 11q13.5 gene may contribute to a T_H_1-dominant immune profile as seen in Crohn disease and chronic atopic eczema lesions rather than the T_H_2-dominant lesions of acute atopic eczema.^24^

In conclusion, although we have replicated the association of rs7927894 with eczema, much further work remains in identifying the causative variant or variants at this locus as well as functional analyses to determine how these sequence changes contribute to the pathogenesis of this complex disease.

**Key messages**

• This replication of a novel variant on chromosome 11q13.5 (rs7927894), carried out in a large, carefully phenotyped collection of pediatric eczema cases, confirms the locus to be of importance.
• The rs7927894 SNP is prevalent in the general population, with a homozygote frequency of 14.5%; these individuals have an estimated OR of 1.63 (95% CI, 1.18-2.24).
• The rs7927894 SNP has an effect that is independent of, and supplementary to, the well established *FLG* null mutations.

## Figures and Tables

**Table:** Results of statistical testing to compare the fit of genotype and allele models of disease

Model	*df*	No. of observations	AIC	BIC	Nested model [AIC, BIC]	*P* value from Wald test from logistic regression comparing model with nested model
rs7927894 (genotype)	3	1505	1924.11	1940.06	rs7927894 (allele) [1922.12, 1932.75]	.95
rs7927894 (allele)	2	1505	1922.12	1932.75		
rs87776 (genotype)	3	1491	1906.99	1922.92	rs87776 (allele) [1906.59, 1917.20]	.22
rs87776 (allele)	2	1491	1906.59	1917.20		
R501X (allele)	2	1496	1795.79	1806.42		
2282del4 (allele)	2	1505	1829.93	1840.56		
R2447X (allele)	2	1497	1891.09	1901.71		
S3247X (genotype)	3	1498	1893.92	1908.86	S3247X (allele) [1892.75, 1903.37]	.34
S3247X (allele)	2	1498	1892.75	1903.37		
Combined *FLG* (genotype)	3	1475	1633.12	1649.01	Combined FLG (allele) [1635.00, 1645.59]	.12
Combined *FLG* (allele)	2	1475	1635.00	1645.59		
rs7927894 (genotype) + R501X (heterozygote) + 2df interaction	6	1488	1789.70	1821.53	rs7927894 (genotype) + R501X (het) [1787.97, 1809.19]	.31
rs7927894 (allele) + R501X (het) + 1df interaction	4	1488	1787.46	1808.68	rs7927894 (allele) + R501X (het) [1785.99, 1801.90]	.47
rs7927894 (genotype) + 2282del4 (het) + 2df interaction	6	1493	1823.15	1855.00	rs7927894 (genotype) + 2282del4 (het) [1819.17, 1840.41]	.99
rs7927894 (allele) + 2282del4 (het) + 1df interaction	4	1493	1819.15	1840.39	rs7927894 (allele) + 2282del4 (het) [1817.17, 1833.10]	.88
rs7927894 (allele) + R2447X (het) + 1df interaction	4	1491	1880.40	1901.63	rs7927894 (allele) + R2447X (het) [1878.64, 1894.56]	.63
rs7927894 (genotype) + S3247X (het) + 2df interaction	6	1490	1882.11	1913.94	rs7927894 (genotype) + S3247X (het) [1878.70, 1899.92]	.74
rs7927894 (allele) + S3247X (het) + 1df interaction	4	1490	1878.68	1899.91	rs7927894 (allele) + S3247X (het)	
rs7927894 (genotype) + *FLG* (het) + 2df interaction	6	1427	1619.32	1650.90	rs7927894 (genotype) + FLG (het) [1615.92, 1636.97]	.74
rs7927894 (allele) + *FLG* (het) + 1df interaction	4	1427	1615.84	1636.89	rs7927894 (allele) + FLG (het) [1613.97, 1629.76]	.71

**Table:** Cross-classification of genotypes used for the interaction analysis

		Controls			Cases		
rs7927894			rs7927894		
AA	Aa	aa	AA	Aa	aa
R501X	AA	335	482	134	114	198	72
	Aa	14	20	10	28	62	19
	aa	0	0	0	0	1	1
2282del4	AA	335	479	138	116	210	77
	Aa	14	22	6	27	52	17
	aa	0	0	0	2	2	2
R2447X	AA	346	497	144	138	248	91
	Aa	3	5	0	6	10	3
	aa	0	0	0	0	0	0
S3247X	AA	343	496	142	136	245	90
	Aa	5	6	2	7	15	3
	aa	1	0	0	0	0	2
Combined *FLG* null genotype	AA	312	448	126	79	138	49
	Aa	36	53	18	47	92	29
	aa	1	0	0	11	21	10

*AA,* Wild-type homozygous individuals for each genetic variant; *Aa,* wild-type/mutant heterozygous individuals; *aa,* individuals who are homozygous for each of the genetic variants tested.

**Table I:** Characterization of 511 Irish pediatric eczema cases

Male sex, n (%)	315/ 511 (61.6)
Age (y), mean (SD)	4.17 (3.96)
No. in age group (%)	
<2 y	201 (39.3)
2-5 y	141 (27.6)
5-10 y	104 (20.4)
10-15 y	55 (10.8)
>15 y	10 (2.0)
Nottingham Eczema Severity Score, mean (SD)	11.29 (4.60)
No. of cases with coexistent asthma (%)	132/511 (25.8)
Serum IgE level (kU/L), mean (SD)	2130.56 (5510.41)

Nottingham Eczema Severity Score, range, 0 to 15, where 9 to 11 defines moderate and 12 to 15 severe disease.^10^

**Table II:** Characterization of Irish control population

Male sex, n (%)	296/1000 (29.6)
Age (y), mean (SD)	33.34 (15.54)
Mean age range in years, n (%)	
<25 y	212 (21.2)
25-50 y	620 (62.0)
>50 y	158 (15.8)

The control population represents 1000 consecutive Trinity Biobank Control samples, derived from Irish adult blood donors.

**Table III:** Genotyping of the 11q13.5 SNP (rs7927894), the *HRNR* SNP (rs877776), and the 4 most prevalent *FLG* null mutations in 511 Irish eczema cases and 1000 individuals from an unselected Irish control population

	rs7927894 (chr 11q13.5)		rs877776 (*HRNR*)		R501X		2282del4		R2447X		S3247X		Combined *FLG* null genotype	
Genotype	Controls	Eczema cases	Controls	Eczema cases	Controls	Eczema cases	Controls	Eczema cases	Controls	Eczema cases	Controls	Eczema cases	Controls	Eczema cases
AA	349	146	688	356	956	385	957	404	992	478	985	472	890	267
Aa	502	266	276	138	44	109	42	96	8	19	13	25	107	168
aa	144	98	26	7	0	2	0	6	0	0	1	2	1	42
Total	995	510	990	501	1000	496	999	506	1000	497	999	499	998	477
Minor allele frequency (%)	39.7	45.3	16.6	15.2	2.2	11.4	2.1	10.7	0.4	1.9	0.8	2.9	5.5	26.4
χ^2^ test from logistic regression (allele model)	*P* = .0025		*P* = .318		*P* = 2.13 × 10^−22^		*P* = 1.53 × 10^−19^		*P* = 1.75 × 10^−4^		*P* = 6.55 × 10^−5^		*P* = 5.25 × 10^−42^	
Fisher exact test (genotype model)	*P* = .010		*P* = .309		*P* = 6.07 × 10^−25^		*P* = 4.90 × 10^−22^		*P* = 9.14 × 10^−5^		*P* = 2.23 × 10^−5^		*P* = 1.26 × 10^−50^	

*AA,* Wild-type homozygous individuals for each genetic variant; *Aa,* wild-type/mutant heterozygous individuals; *aa,* individuals who are homozygous for each of the genetic variants tested. Each of the 2 SNPs and 3 of the 4 *FLG* mutations were in Hardy-Weinberg equilibrium within the control population; S3247X was significantly out of Hardy-Weinberg equilibrium in the control group (*P* = .0001).

**Table IV:** Results of logistic regression analysis to estimate ORs for eczema by using allele and genotype models

		rs7927894 (chr 11q13.5)	rs877776 (*HRNR*)	R501X	2282del4	R2447X	S3247X	Combined *FLG* null genotype
OR calculated using the allele model (95% CI)		1.27 (1.09-1.49)	0.90 (0.73-1.11)	6.20 (4.30-8.96)	5.58 (3.85-8.11)	4.93 (2.14-11.34)	3.52 (1.90-6.52)	5.81 (4.51-7.49)
OR calculated using the genotype model (95% CI)	Heterozygotes vs wild type	1.27 (0.99-1.61)	0.97 (0.76-1.23)	6.15 (4.25-8.90)	5.41 (3.70-7.92)	4.93 (2.14-11.34)	4.01 (2.03-7.91)	5.23 (3.96-6.91)
	Homozygotes vs wild type	1.63 (1.18-2.24)	0.52 (0.22-1.21)	NA	NA	NA	4.17 (0.38-46.14)	140 (19.18-1021)

*NA,* Not analyzed because of absence of homozygote individuals in the cases and/or control group.
